# Vaginal Vault Prolapse

**DOI:** 10.1155/2009/275621

**Published:** 2009-08-11

**Authors:** Azubuike Uzoma, K. A. Farag

**Affiliations:** Barnsley Hospital, NHS Foundation Trust, Gawber Road, Barnsley S75 2EP, UK

## Abstract

*Introduction*. Vaginal vault prolapse is a common complication following vaginal hysterectomy with negative impact on women's quality of life due to associated urinary, anorectal and sexual dysfunction. A clear understanding of the supporting mechanism for the uterus and vagina is important in making the right choice of corrective procedure. Management should be individualised, taking into consideration the surgeon's experience, patients age, comorbidities, previous surgery and sex life. *Result*. Preexisting pelvic floor defect prior to hysterectomy is the single most important risk factor for vault prolapse. Various surgical techniques have been advanced at hysterectomy to prevent vault prolapse. Studies have shown the McCall's culdoplasty under direct visualisation to be superior. Vault prolapse repair rely on either the use of patient's tissue or synthetic materials and can be carried out abdominally or vaginally. Sacrospinous fixation and abdominal sacrocolpopexy are the commonly performed procedures, with literature in favour of abdominal sacrocolpopexy over sacrospinous fixation due to its reported higher success rate of about 90%. Other less commonly performed procedures include uterosacral ligament suspension and illiococcygeal fixation, both of which are equally effective, with the former having a high risk of ureteric injury. Colpoclesis will play a greater role in the future as the aging population increases. Mesh procedures are gaining in popularity, and preliminary data from vaginal mesh procedures is encouraging. Laparoscopic techniques require a high level of skill and experience. There are many controversies on the mechanism of prolapse and management techniques, which we have tried to address in this article. *Conclusion*. As the aging population increases, the incidence of prolapse will also rise, older techniques using native tissue will continue, while new techniques using the mesh needs to be studied further. The later may well be the way forward in future.

## 1. Introduction

Vaginal vault prolapse has been defined by the International Continence Society as descent of the vaginal cuff below a point that is 2 cm less than the total vaginal length above the plane of the hymen [1]. It occurs when the upper vagina bulges into or outside the vagina. 

 Coexistent pelvic floor defects which may be a cystocoele, rectocoele or enterocoele are present in 72% of patients with vault prolapse [2]. Prolapse does have a negative impact on these women's quality of life due to associated urinary, ano-rectal, as well as coital dysfunction. It is therefore important to counsel these women and carefully assess the defects of the various vaginal compartments before planning management. A clear understanding of the supporting mechanisms for the uterus and the vagina is important in order to make the right choice of the corrective procedure and also to minimise the risk of posthysterectomy occurrence of vault prolapse. 

 The surgical options for the correction of vault prolapse lie between the vaginal and the abdominal approach. The choice of procedure should be based on the patient's age, co-morbidity, previous surgery and the level of physical and sexual activity [3]. Also the experience of the surgeon influences the choice of operation. Importantly, greater awareness of the pelvic anatomy and the technique at the time of the original hysterectomy will significantly reduce the incidence of subsequent vault prolapse. 

 The satisfactory correction of vaginal vault prolapse is a formidable surgical challenge and many techniques have been described for the correction of this distressing problem. The aims of prolapse surgery are to restore normal vaginal supports whilst maintaining vaginal capacity and coital function. 

 This article is to review the problem of vault prolapse and the various techniques for its correction, with critical evaluation of their success and possible complications.

## 2. Anatomic Background

The upper vagina, cervix, and uterus are attached to the pelvic sidewalls by broad sheets of endopelvic fascia. These sheets of tissue are usually referred to as the cardinal and utero-sacral ligaments. They originate over the region of the greater sciatic foramen and lateral sacrum, and insert into the side of the cervix as well as the upper one-third of the vagina. Although the cardinal and the utero-sacral ligaments have separate names, they are actually a single unit. The endo-opelvic fascia in this region consists mainly of perivascular collagen and elastin but also contains a considerable amount of nonvascular smooth muscle and the autonomic nerves to the uterus and bladder. Below the level of the uterus, the endo-opelvic fascia attaches the upper one-third of vagina to the pelvic sidewalls in the same way that the cardinal and utero-sacral ligaments provide attachment for the uterine cervix [5]. The middle one-third of the vagina is attached more directly to the lateral pelvic sidewalls by the pubocervical and rectovaginal fasciae, which are nothing more than downward continuations of the cardinal and uterosacral ligaments. These structures attach the lateral margins of the vagina to the pelvic sidewalls on each side, stretching the vagina from one side of the pelvis to the other so that its anterior wall forms a horizontal sheet on which the bladder rests. The posterior attachment of the vagina to the pelvic sidewalls creates a similar sheet that prevents the rectum from prolapsing forward. This is the rectovaginal fascia. 

 On the other hand the muscular levator plate provides indirect support for the upper genital tract by acting as a platform against which the upper vagina and other pelvic viscera are compressed during rises in intraabdominal pressure. The levator plate is formed by the fusion of the right and left bellies of the levator ani muscle behind the rectum and anterior to the coccyx. Sublaxation of the levator plate will cause it to act like a slide, down which the rectum and upper genital tract may descend with rises in intraabdominal pressure. 

 The cardinal and uterosacral ligaments form a complex of visceral supporting tissues to the upper vagina and cervix and, after hysterectomy, to the vaginal cuff. They pull the upper vagina horizontally back toward the sacrum and thus suspended it over the muscular levator plate. Anatomically, the insertion of the cardinal and uterosacral ligaments to the pericervical ring occurs at the level of the ischial spines. Clinically, detachment of the cardinal—uterosacral ligament complexes from pericervical ring occurs at the level of the ischial spines and provides the anatomic rationale for development of uterine descent posthysterectomy, vaginal vault prolapse, and enterocele (apical prolapse).

## 3. Risk Factors

The risk of genital prolapse increases with increasing parity and advancing age. Previous surgery to correct pelvic organ support defects has been consistently identified as risk factors for the development of pelvic organ prolapse. Several other factors have also been implicated, including vaginal versus abdominal delivery for term infant, hysterectomy, congenital defects, races, lifestyle, and chronic disease that increase intrabdominal pressure (e.g., chronic constipation, pulmonary disease, and obesity). However the role of some of these factors is not fully understood.

## 4. Childbirth

Women who had 4 or more vaginal deliveries have 12 times more risk of genital prolapse [6]. From the literature, it appears that vaginal delivery causes damage to the pudendal nerve and promotes the development of pelvic organ prolapse. There are suggestions that instrumental vaginal delivery, especially forceps delivery increase the risk [7]. Also it was demonstrated that Caesarean section can avoid the pudendal nerve damage caused by vaginal delivery [8]. Inspite of the absence damage to the pudendal nerve at caesarean section Maclennan et al. [9] showed that there was no significant difference in pelvic floor dysfunction between caesarean section and vaginal delivery. However pelvic floor dysfunction was significantly commoner following Instrumental delivery.

## 5. Age

Many literatures show increasing prevalence of pelvic organ prolapse in an aged population [10]. It has been shown that there is a 12% increase in the incidence of severe pelvic organ prolapse with each year of advancing ae, or roughly a doubling of the incidence for every decade of life [11].

## 6. Previous Surgery to Correct Pelvic Organ Support Defects

Recurrence rates for surgical correction pf pelvic organ prolapse are in rate 10% to 30% range [10, 12]. By analysing the different risk factors for developing severe pelvic organ prolapse, the previous surgery to correct prolapse was the single greatest risk factor [11]. It appears that pathophysiology of the prolapse is not fully understood and the current practice for surgical correction of prolapse may be inadequate.

## 7. Hysterectomy

There is no consensus on the role of hysterectomy as a cause of subsequent development of pelvic organ prolapse. 

 The incidence of prolapse, which required surgical correction following hysterectomy, is 3.6 per 1000 person-years of risk. The cumulative risk rises from 1% three years after a hysterectomy to 5% 15 years after hysterectomy. Also the risk of prolapse following hysterectomy is 5.5 times in women whose initial hysterectomy was for genital prolapse as opposed to other reasons. Some studies have reported an incidence of up to 43% [3, 13]. 

 Dällenbach et al. conducted a case control study involving 114 women who required pelvic organ prolapse surgery after initial hysterectomy and found that risk factor included preopertional prolapse grade 2 or more, 95% CI 1.3–48.2 and history of vaginal delivery, 95% CI 1.3–19 [14]. Marchionni et al. after following up 2670 women over 9–13-years (mean 11 years) also concluded that incidence of vaginal vault prolapse was low when hysterectomy is performed in the absence of defect in the pelvic support [15]. These support the view that vault prolapse following hysterectomy is more likely if there was pre-existing pelvic floor defect or prolapse.

## 8. Evaluation and Description of Vault Proalpse

Most vaginal cuff prolapses include apical enterocele where the pubocervical and rectovaginal fascia have separated. The peritoneum becomes stretched and comes in direct contact with the vaginal epithelium creating a true hernia. The vaginal epithelium is stretched and becomes very smooth without rugae. There is always some degree of high cystocele formation and high rectocele formation associated with the vaginal vault prolapse. 

 Pelvic Organ Prolapse Quantification (POP-Q) is an objective and standardised system of prolapse classification introduced in 1996, by the International Continence Society. It is a useful tool in assessing the extent of prolapse. It has the added advantage of its use in evaluating surgical and nonsurgical treatment outcomes and for clinical research purposes. 

 The clinical application of 3D-MRI is unclear. A study by Cortes et al. involving 51 women aged 40–95 (mean 64 years) showed poor correlation between MRI and clinical assessment especially in vaginal apical prolapse. 

 However MRI allows identification of other prolapsing compartment and may be complimentary in complex apical prolapsed [16]. 

 Vaginal prolapse is distressing and disabling condition to women. The symptom of feeling “something coming down”, “feeling pressure in the vagina” is always a common complaint. Urinary symptoms, such as poor stream, hesitancy, straining to void, incomplete emptying, recurrent urinary tract infections, and the need to reduce the bulge digitally to void or defecate may also present especially when associated with anterior and posterior compartment prolapse. 

 Asking women about their prolapse symptoms may cause embarrassment and a symptomatic assessment by the physician may be difficult or inaccurate. There are validated, reliable, and easily comprehensible questionnaires designed to assess the severity of symptoms of prolapse and their impact on quality of life. One very useful questionnaire is the prolapse Quality-of-life Questionnaire [17].

## 9. Non Surgical Management

Conservative management will include pelvic floor exercise and pessaries, commonly ring and shelf pessaries. Their role in vault prolapse management is unclear and there is no evidence to suggest that pelvic floor exercise is helpful [18]. However pessaries may have a limited role in the very frail and elderly in whom surgery is not an option.

### 9.1. The Surgical Management (Vaginal Approach)

The overall aim of surgery is to improve the quality of life taking into account the individual peculiarity of each patient. All aspects of the prolapse pathology, patient's lifestyle, age, presence of co-morbidities and sexual function must be taken into consideration. It is also important for the surgeon to understand patient's expectation, discuss available options including their drawbacks so that the appropriate procedure with potential to fulfil her expectations can be achieved. 

 For the patient with good pelvic floor muscle strength as assessed by clinical examination and reasonably substantive endopelvic fascia, a vaginal approach using native tissues may be appropriate. The vagina is anchored to existing stable structures like the sacospinous ligament, illiococcygeous muscle and endopelvic fascia. Women with attenuated fascia, poor pelvic floor muscle strength, repeat repair or severe ongoing physical stress are better served by a technique of vault suspension that provides compensatory repair either through vaginal or abdominal approach using the mesh.

### 9.2. Prevention of Vault Prolapse (McCall Culdoplasty)

It was described by McCall in 1957 as a technique to correct enterocoele [19] and involves the suspension of the vault into the origins of the uterosacral ligaments and obliteration of the cul-de-sac. The original description involved extensive excision of vaginal epithelium, which often resulted in dyspareunia. More recently Elkins et al. described a high McCall culdoplasty [20]. The technique was described to repair the prolapsed vagina at hysterectomy. After the uterine fundus was delivered through an anterior colpotomy incision, the uterosacral ligaments are systematically plicated from the posterior cervix back into the pelvic cavity, until two fingerbreadths remain between the rectum and the plicated ligaments. The main problem with this technique is the risk of ureteric injury. However this can be eliminated by the use of routine cystoscopy with or without methylene blue to identify ureteric efflux following the procedure. Cruikshank and Kovac [4] in a RCT involving 100 patients compared Moschcowit type closure, simple peritoneum closure with McCall culdoplasty. The result (Tables 1  and 2) showed that McCall culdoplasty was more effective than either simple closure of the peritoneum or Moschcowitz over a 3 year follow up in preventing enterocoele. 

Prophylactic McCall Culdoplasty at the time of vaginal hysterectomy for vaginal prolapse is our routine practice, followed by routine cystoscopy to confirm the safety of the ureters.

### 9.3. Sacrospinous Fixation

The procedure was first described by Miyazaki [21] in 1987 and later popularised by Sharp and Richer [6, 22] and by Erata et al. and Lang et al. [23, 24]. It was originally described as a bilateral procedure but subsequently being done as a unilateral procedure. The later results in less tension, though the bilateral technique is more anatomical and maintain a wide vaginal vault. 

The technique comprises dissection into the paravaginal space and the ischial spine is identified. Using a Deschamps ligature carrier, two nonabsorbable sutures are placed through the sacrospinous ligament, one and a half to two fingerbreadths medial to the ischial spine. One end of each suture is attached to the under surface of the posterior vaginal wall at the apical area. When the posterior colporrhaphy reaches the mid-portion of the vagina, the sacrospinous sutures are tied, firmly attaching the vaginal apex to the surface of the coccygeal-sacrospinous ligament complex with no intervening bridge of suture material. Several modifications of this technique were described and mainly involved different methods of placing the sutures into the ligament. The Miyazaki technique uses the Miya hook ligature carrier [25], and the Sharp technique uses the Shutt suture punch system [26]. 

 There are no randomised studies to compare efficacy of either unilateral or bilateral vaginal vault suspension, so there is no evidence to recommend either bilateral or unilateral sacrospinous fixation [27]. 

 In an extensive review of the literature Virtanen and Makinen [28], quoted 18% recurrent prolapse between vault eversion, cystoceles, and rectoceles. More specifically Holley (1995) showed the development of asymptomatic cystocele in 92% [29]. Retrospective study by Colombo et al. involving 124 women who had either sacrospinous fixation or McCall's culdoplasty found no significant difference in the incidence of vaginal vault prolapse after 4 years, but sacrospinous fixation took longer to perform and was associated with more blood loss. Also more women who had sacrospinous fixation, later developed grade 2 or 3 anterior vaginal wall prolapse [30]. It is not an ideal operation for the sexually active woman, as it leads to a less physiological axis than sacrocolpopexy. It is also associated with exaggerated retroversion of the vagina [31]. 

 Although infrequent, haemorrhage is the most common complication but it is rarely of a life threatening nature. It could be a result of injuring the pudendal artery or vein, or the hypogastric venous plexus. 

 Other complications include injury to the bladder or rectum. Transient and self-limiting gluteal pain could result from injuring the small nerve that runs through the coccygeal-sacrospinous ligament complex. Immediate and severe postoperative gluteal pain radiating to the posterior surface of the leg, and often associated with perineal parathesia, indicates posterior cutaneous, pudendal, or sciatic nerve trauma. The recommended treatment for the later is immediate reoperation and releasing the offending suture and repositioning it to a more medial position [32]. In a study by Pollak et al. complication rates were compared for three techniques, namely standard needle driver with direct visualisation, Deschamps ligature carrier by palpation and Miya hook ligature carrier by palpation and the conclusion was that postoperative complications related to suture passage were lower under direct visualisation 2% versus 18%, *P* = .002 [33]. 

 Anterior compartment displacement following this procedure has commonly been reported though debate persists with some surgeons believing that these are actually pre-existing defect missed at initial evaluation. Routine anterior repair at sacrospinous fixation has been suggested by some as a way to address this [34]. This though is not the universal practice. 

 De novo urinary symptoms like overactive bladder or stress incontinence can occur just like in prolapse operations involving the anterior vaginal wall, but in spite of some of these drawbacks, this procedure may be more suitable for the elderly where sexual function is not important. More importantly in these elderly patients with coexisting chronic medical conditions where general anaesthesia is unsuitable or outright dangerous, sacrospinous fixation which can be performed under regional anaesthesia will be the procedure of choice. Success rate of between 75% and 97% has been reported [35].

### 9.4. Iliococcygeal Fixation

The technique was first described by Sze and Karram in 1997 [36] and comprises the fixation of the everted vaginal apex to the ilioccygeal fascia just below the ischial spine. It has subsequently been popularised by Holley et al. [37]. It is usually done as a bilateral procedure as it imposes less tension on the vaginal wall than sacrospinous fixation. The iliococcygeal muscle can be approached through either an anterior or posterior vaginal wall incision. It is relatively easier than sacrospinous fixation and can be done in conjunction with vaginal hysterectomy or as a separate procedure for correction of vault prolapse. A lower rate of postoperative cystocoele, bleeding and pain has been suggested but in a study [38] comparing illiococcygeal fixation to sacrospinous fixation, there was no difference in outcome for postoperative cystocoele, bleeding and pain in both procedures. Success rate were also similar. 

 In a small series Carey reported 11% recurrence following sacrospinous fixation and 14% following iliococcygeal fixation with cystocele being the commonest recurrent prolapse in both groups [39]. 

 There has been suggestion of reduced risk of injury to the pudendal nerves and vessels, and less chance of vaginal shortening, but certainly illiococcygeal fixation offers no additional benefit over sacrospinous fixation. The Royal College of Obstetricians and Gynaecologists (RCOG) in its green-top guideline, no. 46 states “Illiococcygeus fixation does not reduce the incidence of anterior vaginal wall prolapse associated with vaginal sacrospinous fixation and should not be routinely recommended” [40].

### 9.5. Utreosacral Suspension

This has been described as a bilateral procedure carried out vaginally. It can also be carried out via an abdominal or laparoscopic approach. The aim is to place sutures through the uterosacrals at the level of the ischial spine, with one arm brought out through the lateral aspect of the rectovaginal fascia and the other through the pubocervical fascia on each side. These are tied anchoring the vaginal cuff to the uterosacrals. The biggest risk is injury to the ureters (up to 10.9%) due to its proximity to the anterior border of the uterosacrals, especially at the level of the cervix. Other complications include bowel injury, bladder injury, urinary tract infection and blood transfusion. Barber et al. followed up of 46 women, after vaginal uterosacral suspension over a mean period of 15 months and showed 90% had resolution of prolapse symptoms and improvement in the stage of prolapse [41]. Most gynaecologist believe the uterosacral ligaments are compromised in the first place, for prolapse to occur, and for this reason will prefer sacrospinous fixation, while some suggest that the uterosacral ligaments are not weakened, but instead break at specific points resulting in enterocoele and vault prolapse. The later school of thought believe that the uterosacral can be used, even in severe prolapse by identifying the distal portion of breakage and anchoring the vagina high above this point to the uterosacral ligament using an intraperitoneal approach [34].

### 9.6. Infracoccygeal Sling Sacropexy

Petros [42] described this new technique and reports on his experience of the first 75 cases. The principle of this technique is to create artificial uterosacral ligaments by inserting woven nylon tapes along their anatomical path [42]. 

 The technique comprises a transverse incision on the posterior vaginal wall 1.5–2 cm below the hysterectomy scar line and opened anteroposteriorly. The enterocele sac is placed backwards and allowed access to the laterally displaced uterosacral ligaments. At this point the enterocele sac is reduced with a purse string suture. The next step is making bilateral incisions 0.5 cm long in the perianal skin at 4 and 8 o’clock, halfway between the coccyx and external anal sphincter. Having slid the conical head of the tunneller subdermally to the level of 3 and 9 o’clock, the handle is lifted upward 90 degrees so that the head is parallel to the floor. The shaft of the tunneller is then thrust forward into the ischiorectal fossa. This action penetrates the levator plate and brings the conical head into a position behind the uterosacral ligament. Under direct vision, with finger placed in the rectum to locate the position of the rectal wall, the conical tip of the tunneller is gently inclined medially towards the vaginal vault. The tip is then penetrating the fascia adjoining the vagina and rectum. A 6 mm woven nylon tape was threaded into the eye of the plastic insert and brought into the transverse incision. The procedure is repeated on the contralateral side, leaving the tape as a U entirely unfixed at the sacral end. The tape is then sutured to the vault at each corner at the estimated insertion site of the uterosacral ligament. The tape is then gently stretched by pulling on each perineal end, and left entirely free and unfixed. 

 In his series of 75 patients Petros reported 5% recurrence of vault prolapse at a follow up between 1–4 years, 16% de novo anterior wall prolapse, and 4% partial rectocele [42]. Rectal perforation during insertion of the tape was reported in 2 patients, identified during the procedure and had no long-term sequel. Current evidence on the efficacy and safety of this procedure is inadequate. Few clinical data are available on the success rate of this procedure, though a recent report quoted a 75% improvement in vault prolapse [43]. 

 The National Institute for Health and Clinical Excellence recommends that this procedure should only be used with special arrangement in a clinical governance or research setting [44].

## 10. Abdominal Approach

### 10.1. Abdominal Sacrocolpopexy

Abdominal sacrocolpopexy, employing retroperitoneal interposition of a suspensory synthetic, autologous or allograft prosthesis between the vaginal vault and the sacral promontory was first described by Lane in 1962 [45]. This method has proven to be superior to other surgical techniques in terms of restoration of the normal vaginal axis and maintenance of vaginal capacity [46, 47]. Although the short-term success rates reported for this procedure are in excess of 90%, the long-term outcome remains unclear and significant complications include postoperative stress urinary incontinence, dyspareunia and erosion of synthetic graft material. 

 The abdominal sacral colpopexy employs the interposition of a synthetic mesh or tissue graft between the vagina and sacrum. This technique allows for more global support of the vagina and distribution of tension over a larger surface area. Previous authors have reported severe and occasionally life-threatening haemorrhage from the preexistingsacral vessels, when sutures were placed in the hollow of the sacrum [48, 49]. To reduce this risk the operative technique was therefore modified and sutures placed more proximally over the sacral promontory. Contrary to previous reports, the point of sacral attachment does not affect the vaginal axis and attachment to the sacral promontory allows effective restoration of vaginal support, while maintaining both vaginal capacity and coital function [50, 51]. Most surgeons will bury the mesh under the peritoneum to avoid bowel erosion, while some do not and others will tunnel the mesh from the vaginal vault to the sacral promontory without dissecting the peritoneum. Which method is best is still controversial. Different methods of mesh attachment to the vagina have been described and to date these remain very controversial. These include attaching a full length of mesh to the whole length of the rectovaginal septum. Another method involves a double attachment of the mesh to the anterior and posterior vaginal surfaces with reported good results. There are usually other associated defects like anterior or posterior vaginal wall defect in varying degrees with divided opinion and debate amongst surgeons on completing it either vaginally or abdominally [34]. There is no simple answer, but every patient has to be considered individually and the associated defects assessed properly, so that a clear plan of surgical repair can be agreed with the patient bearing in mind other factors like coital function. 

 Consistent cure rate of more than 90% has been reported [52], with some studies reporting up to 95% [53]. 

 Mesh erosion following the use of polypropylene graft was reported to complicate 2–2.7% of cases [54, 55]. This will necessitate revision or removal of the mesh. In most of the cases, this occurs at the vaginal vault resulting in dyspareunia and vaginal discharge within the first six months. Mesh erosion is usually predisposed to by marked scarring and thinning of the vagina from previous vaginal repairs or a combined abdominal hysterectomy and sacral colpopexy. This problem can be eliminated by the use of donor fascia lata or a xenograft. 

 This procedure has added advantage over the traditional procedures because it maintains the normal axis of the vagina, with preservation of maximal vaginal length which is desirable for optimal sexual function. It also provides a source of strength in patients with weak tissue or recurrent prolapse [56]. For these reasons it is quite a fairly common operation with 38% of surgeons in a national survey carrying it out for vault prolapse [57]. It is further associated with a lower rate of recurrent prolapse and dyspareunia [58] which makes it popular choice amongst surgeons especially in fit patients. Part of its drawback includes the fact that it is performed via laparotomy with all the associated risk of internal organ injury, longer operation time and hospital stay, so these need to be balanced against the benefits. In the very elderly with coexisting medical pathology, the risk of laparotomy coupled with the extra risk of general anaesthesia will make this procedure unsuitable [56].

## 11. Laparoscopic Approach

### 11.1. Laparoscopic Sacral Colpopexy

In theory laparoscopic approach to the repair of the vault prolapse should follow the same principle as in the open technique, with laparoscopy only being the mode of surgical access. However a highly skilled and experienced laparoscopic surgeon is crucial. This approach has a steep learning curve and takes many years of practise to acquire the necessary skills. 

 In a small study by Hsiao et al. in 2004, comparing laparoscopic sacrocolpopexy (25 patients) and abdominal sacrocolpopexy (22 patients), it showed that blood loss and hospital stay was significantly less in the laparoscopic group (*P* = .002), though operation time was longer (*P* < .001). However there was no difference in efficacy of both methods. Success rates of 95% for abdominal and 100% for laparoscopic techniques has been reported [53].

### 11.2. Laparoscopic Uterosacral-Ligament Vault Suspension

The technique begins with the identification of the vaginal vault apex, and the rectovaginal and pubocervical fascia facilitated by the use of a vaginal probe. Traction is placed on the vaginal probe forward to stretch the utero-sacral ligaments so they can be identified and traced backwards. At this stage both ureters are identified. The peritoneum overlying the vaginal apex is incised to expose the pubocervical fascia anteriorly and the rectovaginal fascia posteriorly. The rest of the procedure will follow along the same steps for the open technique, and the uterosacral ligament on each side is attached using nonabsorbable sutures to the ipsilateral side of the vaginal vault. Intra- or extracorporeal knots can be used depending on the surgeon's preference. 

 There is a high risk of ureteric injury, so cystoscopy is advised after suture placement. Success rate of up to 90% over a 2 year period has been reported [59]. 

 Laparoscopic surgery has a steep learning curve and not all surgeons will have the necessary skill to excel especially considering the technical difficulty and longer operation time. The main advantage is good exposure of the operation field enabling the surgeon to fully evaluate and treat other components of the prolapse effectively. Most recently new innovations like robotics, though in its infancy is helping to address some of the limitations of laparoscopy by providing better technical features such as 3D vision and more precise robotic instrument manoeuvrability. One of the latest and increasingly popular systems with varied application in different specialities, the Da Vinci system is at the fore of this new frontier [60].

### 11.3. Colpocleisis

This is a procedure that may gain in popularity in the coming years and as life expectancy rises in an aging population. 

 Colpocleisis involves surgical obliteration of the lumen of the vagina. Different methods are described including purse-string closure, vaginectomy and in association with other continence procedures [40]. Basically the vaginal epithelium is mobilised anteriorly and posteriorly leaving about 2 cm from the vault above and also from the urethral meatus below. The prolapse is reduced by placing progressive sutures anteroposteriorly, till the prolapsed tissues are above the level of the levator plate. It can also be carried out as a partial or total procedure. The partial procedure is usually reserved for women with an intact prolapsed uterus with the aim of giving access to any discharge or bleeding from the uterus via a small opening. 

 It is suitable for the frail elderly woman who is not sexually active and for whom conservative methods like the pessary is not ideal. It has the advantage that it can also be carried out under local anaesthesia and involves a shorter operation time. Essentially it is about improving the quality of life. In one of the largest case series involving 41 women carried out at Temple University Philadelphia between November 1994 and June 2001, there was only one case of bladder injury, 2 cases of self limiting rectal bleed and average hospital stay of 2 days [61]. 

De novo urinary stress incontinence of up to 27% in previously continent women has been reported [62], though no intraoperative complication has been reported in the literature [63]. 

 Success rate of 97% and above have also been reported [64].

## 12. Vaginal versus Abdominal Approach

A prospective randomised study [12] compared vaginal (bilateral sacrospinous vault suspension and paravaginal repair) versus abdominal (colposacral suspension and paravaginal repair). 

 These patients were followed up for up to 5 years. Table 3 represents the outcome.

This study did show that the vaginal route had significantly higher postoperative incontinence rate, longer period of catheter use and recurrence was less likely to occur following the abdominal route. However there were more transfusions and febrile morbidity in the abdominal group, though not significant. This study also noted a higher rate of reoperation for recurrent prolapse, 33% vaginal versus abdominal 16% confirming that the abdominal route has a better success rate.

## 13. Role of Mesh in Vault Prolapse

Synthetic mesh has been commonly used to manage pelvic organ and prolapse, even though the more traditional suture repair technique is still the primary choice, with the mesh mainly reserved for repeat procedures and large defects. 

 A multicentric retrospective study [65] involving 110 patients, of which 59 had total mesh repair, (transvaginal) using the prolift (Gynaecare) system, showed a recurrence rate of 4.75% at 3 month follow up. In spite of the short follow-up in this study, the total mesh may possibly address the issue of high rate of recurrence commoner with the more traditional methods. Other types of mesh like the Apogee (posterior vaginal wall) and Perigee (anterior vaginal wall) have been used for management of recurrent cystocoele and rectocoele with or without vault prolapse. Success rate of 93% has been reported [66]. However there are no randomised controlled studies to compare this procedure with abdominal sacrocolpopexy or uterosacral suspension for now [67].

## 14. Conclusion

Vault prolapse repair is based on use of native tissues or synthetic materials. There is no consensus on the mechanism and management of vault prolapse, but what is accepted by all is the need to properly assess these patients, involve them in the management and to agree on the type of surgery that will be suitable for their own peculiar circumstance. The mesh is gaining in popularity, but there are no studies yet on its long term efficacy though initial results are very encouraging.

## Figures and Tables

**Table 1:** RCT involving 100 patients comparing procedures used at the time of hysterectomy to prevent enterocoele [4].

Type of procedure	No. of patients	Prolapse rate over 3-years
Moschcowit type closure	33	30.3%
Modified McCall culdoplasty	33	6.1%
Simple closure of peritoneum	34	39.4%

**Table 2:** Incidence and stages of enterocoele at follow-up [4].

Type	1 year	2 years	3-years
McCall culdoplasty (*n* = 33)	Stage 1 = 0	Stage 1 = 2	Stage 1 = 2
	Stage 2 = 0	Stage 2 = 0	Stage 2 = 0
			(*P* = .004)

Moschcowitz (*n* = 33)	Stage 1 = 3	Stage 1 = 3	Stage 1 = 4
	Stage 2 = 0	Stage 2 = 2	Stage 2 = 6

Simple closure of peritoneum (*n* = 34)	Stage 1 = 4	Stage 1 = 5	Stage 1 = 8
	Stage 2 = 4	Stage 2 = 4	Stage 2 = 5

**Table 3:** 

	Vaginal (*n* = 42)	Abdominal (*n* = 38)
Hb change g/dl	2.6 ± 1	3 ± 1
Number transfused	0	2
Dyspareunia	15%	0%
	(Sexually active *n* = 26)	(Sexually active *n* = 15)
Febrile morbidity	4%	8%
Incontinence	44%	23% (*P* < .05)
Catheter duration >5 days	75%	48% (*P* < .05)
Time of recurrence in months	11.2 ± 11.5	22.1 ± 16.2 (*P* < .05)

## References

[1] Abrams P, Cardozo L, Fall M (2002). The standardisation of terminology of lower urinary tract function: report from the standardisation sub-committee of the international continence society. *Neurourology and Urodynamics*.

[2] Sederl J (1958). Zur operation des prolapses der blind endigenden sheiden. *Geburtshilfe Frauenheilkd*.

[3] Flynn BJ, Webster GD (2002). Surgical management of the apical vaginal defect. *Current Opinion in Urology*.

[4] Cruikshank SH, Kovac SR (1999). Randomized comparison of three surgical methods used at the time of vaginal hysterectomy to prevent posterior enterocele. *American Journal of Obstetrics & Gynecology*.

[5] Richer K, Albright W (1981). Long term results following fixation of the vagina on the sacrospinous ligament by the vaginal route. *American Journal of Obstetrics & Gynecology*.

[6] Richter K (1982). Massive eversion of the vagina: pathogenesis, diagnosis, and therapy of the “true” prolapse of the vaginal stump. *Clinical Obstetrics and Gynecology*.

[7] Moalli PA, Ivy SJ, Meyn LA, Zyczynski HM (2003). Risk factors associated with pelvic floor disorders in women undergoing surgical repair. *Obstetrics and Gynecology*.

[8] Snooks SJ, Swash M, Henry MM, Setchell M (1986). Risk factors in childbirth causing damage to the pelvic floor innervation. *International Journal of Colorectal Disease*.

[9] MacLennan AH, Taylor AW, Wilson DH, Wilson D (2000). The prevalence of pelvic floor disorders and their relationship to gender, age, parity and mode of delivery. *British Journal of Obstetrics and Gynaecology*.

[10] Olsen AL, Smith VJ, Bergstrom JO, Colling JC, Clark AL (1997). Epidemiology of surgically managed pelvic organ prolapse and urinary incontinence. *Obstetrics and Gynecology*.

[11] Swift SE, Pound T, Dias JK (2001). Case-control study of etiologic factors in the development of severe pelvic organ prolapse. *International Urogynecology Journal and Pelvic Floor Dysfunction*.

[12] Benson JT, Lucente V, McClellan E (1996). Vaginal versus abdominal reconstructive surgery for the treatment of pelvic support defects: a prospective randomized study with long-term outcome evaluation. *American Journal of Obstetrics & Gynecology*.

[13] DeLancey JOL (1992). Anatomic aspects of vaginal eversion after hysterectomy. *American Journal of Obstetrics & Gynecology*.

[14] Dällenbach P, Kaelin-Gambirasio I, Dubuisson J-B, Boulvain M (2007). Risk factors for pelvic organ prolapse repair after hysterectomy. *Obstetrics and Gynecology*.

[15] Marchionni M, Bracco GL, Checcucci V (1999). True incidence of vaginal vault prolapse: thirteen years of experience. *Journal of Reproductive Medicine for the Obstetrician and Gynecologist*.

[16] Cortes E, Reid WMN, Singh K, Berger L (2004). Clinical examination and dynamic magnetic resonance imaging in vaginal vault prolapse. *Obstetrics and Gynecology*.

[17] Digesu GA, Khullar V, Cardozo L (2000). P-QoL: a validated quality of life questionnaire for the symptomatic assessment of women with uterovaginal prolapse. *International Urogynecology Journal*.

[18] Hagen S, Stark D, Maher C, Adams E (2006). Conservative management of pelvic organ prolapse in women. *Cochrane Database of Systematic Reviews*.

[19] McCall ML (1957). Posterior culdoplasty: surgical correction of enterocele during vaginal hysterectomy: a preliminary report. *American Journal of Obstetrics and Gynaecology*.

[20] Elkins TE, Hopper JB, Goodfellow K, Gasser R, Nolan TE, Schexnayder MC (1995). Initial report of anatomic and clinical comparison of the sacrospinous ligament fixation to the high McCall culdoplasty for vaginal cuff fixation at hysterectomy for uterine prolapse. *Journal of Pelvic Surgery*.

[21] Miyazaki FS (1987). Miya Hook ligature carrier for sacrospinous ligament suspension. *Obstetrics and Gynecology*.

[22] Sharp TR (1993). Sacrospinous suspension made easy. *Obstetrics and Gynecology*.

[23] Erata YE, Kilic B, Güçlü S, Saygili U, Uslu T (2002). Risk factors for pelvic surgery. *Archives of Gynecology and Obstetrics*.

[24] Lang JH, Zhu L, Sun ZJ, Chen J (2003). Estrogen levels and estrogen receptors in patients with stress urinary incontinence and pelvic organ prolapse. *International Journal of Gynecology and Obstetrics*.

[25] Sang WB, Byung HC, Jeong YK, Ki HP (2002). Pelvic organ prolapse and connective tissue abnormalities in Korean women. *The Journal of Reproductive Medicine*.

[26] Cruikshank SH (1991). Sacrospinous fixation—sould this be performed at the time of vaginal hysterectomy?. *American Journal of Obstetrics & Gynecology*.

[27] Randall CL, Nichols DH (1971). Surgical treatment of vaginal inversion. *Obstetrics and Gynecology*.

[28] Virtanen HS, Makinen J (1993). Retrospective analysis of 711 patients operated on for pelvic relaxation in 1983–1989. *International Journal of Gynecology and Obstetrics*.

[29] Inmon WB (1963). Pelvic relaxation and repair including prolapse of vagina following hysterectomy. *Southern Medical Journal*.

[30] Colombo M, Milani R (1998). Sacrospinous ligament fixation and modified McCall culdoplasty during vaginal hysterectomy for advanced uterovaginal prolapse. *American Journal of Obstetrics & Gynecology*.

[31] Rane A, Lim YN, Withey G, Muller R (2004). Magnetic resonance imaging findings following three different vaginal vault prolapse repair procedures: a randomised study. *Australian and New Zealand Journal of Obstetrics and Gynaecology*.

[32] Shull BL, Capen CV, Riggs MW, Kuehl TJ (1993). Bilateral attachment of the vaginal cuff to iliococcygeus fascia: an effective method of cuff suspension. *American Journal of Obstetrics & Gynecology*.

[33] Pollak J, Takacs P, Medina C (2007). Complications of three sacrospinous ligament fixation techniques. *International Journal of Gynecology and Obstetrics*.

[34] Arbel R, Lavy Y (2005). Vaginal vault prolapse: choice of operation. *Best Practice & Research Clinical Obstetrics & Gynaecology*.

[35] Lovatsis D, Drutz HP (2003). Vaginal surgical approach to vaginal vault prolapse: considerations of anatomic correction and safety. *Current Opinion in Obstetrics and Gynecology*.

[36] Sze EHM, Karram MM (1997). Transvaginal repair of vault prolapse: a review. *Obstetrics and Gynecology*.

[37] Holley RL, Varner RE, Gleason BP, Apffel L, Scott S (1995). Recurrent pelvic support defects after sacrospinous ligament fixation for vaginal vault prolapse. *Journal of the American College of Surgeons*.

[38] Maher CF, Murray CJ, Carey MP, Dwyer PL, Ugoni AM (2001). Iliococcygeus or sacrospinous fixation for vaginal vault prolapse. *Obstetrics and Gynecology*.

[39] Carey MP (2003). Pelvic floor disorders symposium; ‘Pelvic Organ Prolapse’. *Australian & New Zealand Continence Journal*.

[41] Barber MD, Visco AG, Weidner AC, Amundsen CL, Bump RC (2000). Bilateral uterosacral ligament vaginal vault suspension with site-specific endopelvic fascia defect repair for treatment of pelvic organ prolapse. *American Journal of Obstetrics & Gynecology*.

[42] Petros PEP (2001). Vault prolapse II: restoration of dynamic vaginal supports by infracoccygeal sacropexy, an axial day-case vaginal procedure. *International Urogynecology Journal and Pelvic Floor Dysfunction*.

[43] Jordaan D-J, Prollius A, Cronjé HS, Nel M (2006). Posterior intravaginal slingplasty for vaginal prolapse. *International Urogynecology Journal and Pelvic Floor Dysfunction*.

[44] National Institute for Health and Clinical Excellence

[45] Lane FE (1962). Repair of posthysterectomy vaginal vault prolapse. *Obstetrics & Gynecology*.

[46] Benson JT, McClellan E (1993). The effect of vaginal dissection on the pudendal nerve. *Obstetrics and Gynecology*.

[47] Grunberger W, Grunberger V, Wierrani F (1994). Pelvic promontory fixation of the vaginal vault in sixty-two patients with prolapse after hysterectomy. *Journal of the American College of Surgeons*.

[48] Sutton GP, Addison WA, Livengood CH, Hammond CB (1981). Life-threatening hemorrhage complicating sacral colpopexy. *American Journal of Obstetrics & Gynecology*.

[49] Iglesia CB, Fenner DE, Brubaker L (1997). The use of mesh in gynecologic surgery. *International Urogynecology Journal and Pelvic Floor Dysfunction*.

[50] Snyder TE, Krantz KE (1991). Abdominal-retroperitoneal sacral colpopexy for the correction of vaginal prolapse. *Obstetrics and Gynecology*.

[51] Fox SD, Stanton SL (2000). Vault prolapse and rectocele: assessment of repair using sacrocolpopexy with mesh interposition. *British Journal of Obstetrics and Gynaecology*.

[52] Addison WA, Bump RC, Cundiff GW (1996). Sacral colpopexy is the preferred treatment for vaginal vault prolapse in selected patients. *J Gynecol Tech*.

[53] Hsiao KC, Latchamsetty K, Govier FE, Kozlowski P, Kobashi KC (2007). Comparison of laparoscopic and abdominal sacrocolpopexy for the treatment of vaginal vault prolapse. *Journal of Endourology*.

[54] Iglesia CB, Fenner DE, Brubaker L (1997). The use of mesh in gynecologic surgery. *International Urogynecology Journal and Pelvic Floor Dysfunction*.

[55] Fynes M, Goh J, Chong C Abdominal Sacral Colpopexy for Vaginal Vault Prolapse, Peri-operative Morbidity and Outcome.

[56] Elliott DS, Krambeck AE, Chow GK (2006). Long-term results of robotic assisted laparoscopic sacrocolpopexy for the treatment of high grade vaginal vault prolapse. *Journal of Urology*.

[57] Jha S, Moran PA (2007). National survey on the management of prolapse in the UK. *Neurourology and Urodynamics*.

[58] Maher C, Baessler K, Glazener CMA, Adams EJ, Hagen S (2007). Surgical management of pelvic organ prolapse in women. *Cochrane Database of Systematic Reviews*.

[59] Carter JE, Winter M, Mendehlsohn S, Saye W, Richardson AC (2001). Vaginal vault suspension and enterocele repair by Richardson-Saye laparoscopic technique: description of training technique and results. *Journal of the Society of Laparoendoscopic Surgeons*.

[60] Hubens G, Coveliers H, Balliu L, Ruppert M, Vaneerdeweg W (2003). A performance study comparing manual and robotically assisted laparoscopic surgery using the Da Vinci system. *Surgical Endoscopy and Other Interventional Techniques*.

[61] Nancy Walsh Colpocleisis seen as effective for prolapse in elderly.

[62] FitzGerald MP, Brubaker L (2003). Colpocleisis and urinary incontinence. *American Journal of Obstetrics & Gynecology*.

[63] Latthe PM, Kamakshi K, Arunkalaivanan AS (2008). Colpocleisis revisited. *The Obstetrician & Gynaecologist*.

[64] Cespedes RD, Winters JC, Ferguson KH (2001). Colpocleisis for the treatment of vaginal vault prolapse. *Techniques in Urology*.

[65] Fatton B, Amblard J, Debodinance P, Cosson M, Jacquetin B (2007). Transvaginal repair of genital prolapse: preliminary results of a new tension-free vaginal mesh (Prolift^TM^ technique)—a case series multicentric study. *International Urogynecology Journal and Pelvic Floor Dysfunction*.

[66] Gauruder-Burmester A, Koutouzidou P, Rohne J, Gronewold M, Tunn R (2007). Follow-up after polypropylene mesh repair of anterior and posterior compartments in patients with recurrent prolapse. *International Urogynecology Journal and Pelvic Floor Dysfunction*.

[67] South MM, Amundsen LC (2007). Pelvic surgery. *OBG Management*.

